# Coverage and Acceptability of Mobile Phone Messages for Cancer Prevention: a Population-Based Study in a Latin American Country

**DOI:** 10.1007/s13187-020-01912-0

**Published:** 2020-11-13

**Authors:** Raúl Murillo, Camila Ordóñez-Reyes, María Caicedo-Martínez, Sandra Paola Vargas, Elsa Ariza, Joachim Schüz, Carolina Espina

**Affiliations:** 1 Centro Javeriano de Oncología, Hospital Universitario San Ignacio, Bogotá, Colombia; 2 Facultad de Medicina, Pontificia Universidad Javeriana, Bogotá, Colombia; 3 Prevention and Implementation Group, International Agency for Research on Cancer, Lyon, France; 4 Bogotá, Colombia; 5 Gerencia de Prestación de Servicios - Nueva EPS, Bogotá, Colombia; 6 Section of Environment and Radiation, International Agency for Research on Cancer, Lyon, France

**Keywords:** Cancer prevention, Health communication, Cell phones, Latin America, m-health, Neoplasms, Colombia

## Abstract

Mobile health (m-health) has shown positive effects on disease prevention; however, several factors might influence its effectiveness, particularly in low- and middle-income countries. Randomized trials provide data with high internal validity but no major information on population impact. We conducted a pilot population-based study to assess the feasibility of cancer prevention through m-health in a Latin American population. A sample of affiliates to a health insurance company in Colombia was randomly selected and assigned to receive a short message service (SMS) or voice messages (VMS) during 4 weeks; weekly frequencies 2 and 7. Baseline and post-intervention surveys were conducted. Overall, 797 affiliates were contacted (SMS 393, VMS 404) but only 15.3% and 24.8% enrolled, respectively. Over 80% acceptability was observed among participants for all items evaluated (usefulness, understandability, timing, and frequency); however, 2-VMS per week was the only frequency consistent with the declared number of messages received and listened. Other frequencies resulted in high reception recall but low willingness to read/listen the messages. The willingness to be part of future programs was 20.0%. The gap between declared acceptability and practice, low participation rates, and low willingness to read/listen messages indicate m-health should be part of multicomponent interventions and should not be conceived as the sole intervention.

## Introduction

Almost seven and a half billion people (98% of the total world population) currently live in areas covered by mobile phone networks, where broadband (3G or higher) reaches 94% of the population [1]. Mobile phones have become the most accessible form of communication in history, with more than eight billion mobile phone subscribers around the world in 2019, more than one billion in the Americas region [1], and mobile technology being the fastest growing communication industry in low- and middle-income countries [2].

The short message service (SMS) is the most used mobile interpersonal communication channel with about 75% of mobile phone users [3]. m-health (mobile health) and particularly multifaceted mobile technology messaging interventions have shown positive effects on different health outcomes such as adherence to prescribed medications, prevention of mother-to-child transmission of HIV, prevention and management of type 2 diabetes, uptake of sexual health services, treatment of alcohol dependency, smoking cessation, and weight loss [4–8].

Besides the effect on specific populations and health conditions, given the global reach of mobile phones and text messages in diverse audiences, interventions based on mobile phone messages aimed at behavioral change could have great impact at the population level. Indeed, a review found positive effects on healthy behaviors across age, minority status, and nationality [9]. However, several factors might influence effectiveness of mobile technology interventions including individual-associated factors (sociodemographics), technology-associated factors (platforms), and contextual factors [9–11].

Regarding the latter, a review of mobile health in low- and middle-income countries found greater number of studies in Africa and Asia than in Latin America, and most studies aimed at controlling infectious diseases and improve treatment adherence [10]. Some of the studies oriented to preventive interventions (mostly around infectious diseases) identified as barriers for implementing m-health in these settings the lack of access to the technology, limitations by type of equipment, and the literacy and capability to use the technology for m-health purposes. Nevertheless, m-health interventions in underserved populations from high-income countries have shown to encourage healthy behaviors, support self-care, and reduce the number of health care visits if messages are designed in agreement with literacy, cultural, and motivational needs [12].

In general, prevention of cancer and chronic diseases has not been a topic of major activity regarding the use of m-health in low- and middle-income countries [10]. In addition, most evidence in the field derives from randomized controlled trials, which provide good-quality data with high internal validity about efficacy of interventions but no major information on the impact of m-health at the population level, since efficacy should be accompanied with good coverage, acceptability, and adherence to reach objectives of massive dissemination. In this regard, population-based studies from low- or middle-income countries (LMIC) have shown high acceptability in surveys (about 87% routine use of mobile phones and 92% acceptability of receiving messages with health advise) [13], but low coverage in studies evaluating preventive interventions (response rate between 1 and 50%, and among responders 4.6% to 74% adherence to follow-up) [14, 15].

To assess the feasibility of cancer prevention interventions through mobile phone messages in a Latin American population, this study assessed the suitability of the strategy (coverage and acceptability), including specific characteristics of the intervention, such as frequency and timing of messages, the influence of mobile technology (SMS or voice messages), and the willingness to use complementary communication modalities (web page).

## Methods

A pilot study was carried out in Colombia among affiliates to one of the biggest health insurance companies in the country. The Colombian health system is individual insurance-based with insurance coverage over 90% and insurance managed by health insurance companies designated by the government through two main insurance plans: payroll system for workers and their families and subsidized health insurance for low-income families. The health insurance company in the study accounts with both health insurance plans. The Ethical Committee at the San Ignacio University Hospital approved the protocol, and informed consent was obtained for respondent participants.

The study had two arms randomly allocated to text (SMS) and voice messages. A second randomization was done independently for every arm to the reception of two versus seven messages per week. Randomization was done by generating random numbers in a computer algorithm without participation of the research team. Inclusion criteria were age 18 years or older and affiliation to the health insurance company selected for the study.

The recruitment was done by phone call with prior authorization of the health insurance company for both insurance plans, and ensuring voluntary participation. Three attempts were done before considering no-response. Participants were given an explanation of the study including the random allocation to the type of messages to receive.

Then, a 4-week intervention was carried out by sending SMS and voice messages with selected cancer prevention recommendations from the European Code Against Cancer [16] (Appendix), with frequency as indicated for each arm in the study and schedule from 8:00 am to 6:00 pm. The messages were complemented with an invitation to visit a website where expanded information on the recommendations was available. SMS were sent through the regular platform of the health insurance company whereas a different dedicated company worked voice messages given the absence of a platform for this service in the health insurance company. A baseline survey on sociodemographics was conducted.

A post-intervention telephone survey was carried out asking the number of messages received during the last week, the number of messages read/listened, the understandability of the messages, the overall perception on usefulness of messages, and suitability of timing and frequency of messages. We also recruited data about visits to the website as it was systematically recommended in the messages.

### Statistical Analysis

The study does not intend to demonstrate differences in response rates between arms; however, differences in participation rates were analyzed. The sample size was estimated independently for each arm for an infinite population with 50% response rate, 95% confidence, and 5% precision (383 individuals per arm). Given that response rates are an outcome, no replacement or losses to follow-up were estimated. Participation data will be useful for recruitment estimates in future studies; therefore, information on sociodemographic characteristics for all invitees was collected.

Participation rates were defined as willingness to receive messages among responders to the call (final estimate combine response to the call and positive answer to the invitation). A structured questionnaire was applied asking for the reception of messages (number of messages received), level of participation (number of messages read/listened), and search for additional information (visit to the website). Preferences of participants on characteristics of messages, including frequency and duration, were investigated.

Response rates were measured as relative frequencies, and association (significance *p* < 0.1) with sociodemographic characteristics evaluated (age, sex, education, and income) by using chi-square tests for categorical covariates and *t* tests for continuous covariates in the univariate analysis. Variables with significant association were included in a logistic regression model defining significant association with *p* < 0.05.

## Results

Overall, 797 affiliates to the insurance company were randomly allocated to the SMS or voice messages groups (393 and 404, respectively). The second randomization yields 195 affiliates in the arm with 2 SMS per week (2-SMS), 198 in the arm with 7 SMS per week (7-SMS), and 202 in the arms with 2 and 7 voice message services per week (2-VMS and 7-VMS).

The mean age was 46.7 years old, 50.8% were men, and 68.5% correspond to the main health insurance plan in the country (payroll system for workers and their families) while 31.5% correspond to the subsidized health insurance plan for low-income families (Table 1). No significant differences were observed between arms. The random selection resulted in affiliates from the 32 administrative departments existing in the country, thus representing all major geographic regions.

**Table 1:** Baseline characteristics of the whole study population

Characteristics	2-SMS (*n* = 195)	7-SMS (*n* = 198)	Total SMS (n = 393)	2-VMS (*n* = 202)	7-VMS (*n* = 202)	Total VMS (*n* = 404)	*p* value
Age							
Mean	47.2	48.3	47.7	46.4	45.2	45.8	0.13
Gender (%)							
Male	99 (50.8%)	99 (50.0%)	198 (50.4%)	103 (51.0%)	104 (51.5%)	207 (51.2%)	0.86
Female	96 (49.2%)	99 (50.0%)	195 (49.6%)	99 (49.0%)	98 (48.5%)	197 (48.8%)	
Health insurance plan (%)							
Subsidized	67 (34.4%)	58 (29.3%)	125 (31.8%)	69 (34.2%)	57 (28.2%)	126 (31.2%)	0.88
Payroll	128 (65.6%)	140 (70.7%)	268 (68.2%)	133 (65.8%)	145 (71.8%)	278 (68.8%)	
Education (%)							
Elementary or lower	155 (79.4%)	156 (78.9%)	311 (79.1%)	160 (79.2%)	152 (75.2%)	312 (77.2%)	0.46
Secondary	22 (11.3%)	21 (10.6%)	43(10.9%)	27 (13.4%)	32 (15.8%)	59 (14.6%)	
Technician	10 (5.1%)	14 (7.1%)	24 (6.1%)	11 (5.4%)	12 (5.9%)	23 (5.7%)	
University	8 (4.1%)	7 (3.5%)	15(3.8%)	4 (2.0%)	6 (3.0%)	10 (2.5%)	
Geographic region (%)							
Andean	101 (51.8%)	92 (46.5%)	193 (49.1%)	108 (53.5%)	107 (53.0%)	215 (53.2%)	0.60
Caribbean	43 (22.1%)	43 (21.7%)	86 (21.9%)	41 (20.3%)	45 (22.3%)	86 (21.3%)	
Pacific	29 (14.9%)	33 (16.7%)	62 (15.8%)	33 (16.3%)	25 (12.4%)	58 (14.4%)	
Orinoquia and Amazonia	22 (11.3%)	30 (15.2%)	52 (13.2%)	20 (9.9%)	25 (12.4%)	45 (11.1%)	

*SMS* short message service (text), *VMS* voice message service, *2-SMS* two SMS weekly, *7-SMS* seven SMS weekly, *2-VMS* two VMS weekly, *7-VMS* seven VMS weekly

A total of 113 (28.7%, 95% CI 24.3–33.5) affiliates in the SMS arm and 239 (59.2%, 95% CI 54.2–64.0) in the VMS arm answered the call for recruitment. Only 60 affiliates from the SMS group (15.3% of the SMS-allocated participants and 53.1% among responders) and 100 affiliates from the VMS group (24.8% of the VMS-allocated participants and 46.5% among responders) accepted the reception of messages and participation in the study (SMS/VMS *p* value *<* 0.01 for overall participation and 0.26 for participation among responders). Among responders to the recruitment call, no differences were observed in age, sex, and health insurance plan between affiliates who accepted the reception of messages and those who did not. Similarly, no differences were observed between responders and no-responders to the recruitment call in age or health insurance plan (*p* > 0.05); however, no-responders were more frequently men (46.0% responders and 54.6% no-responders, *p* = 0.02).

Participants who accepted the reception of messages in the SMS arm showed a higher level of education (Technician/University) than participants who accepted the reception of messages in the VMS arm (*p* = 0.004). No other differences were observed between arms among participants who accepted the reception of messages (Table 2). The multivariate analysis revealed no associations with response to the recruitment call or participation, neither for the general population nor for the VMS arm. In the SMS arm, affiliates from the Caribbean region more frequently participated with borderline significance (*p* = 0.047).

**Table 2:** Baseline characteristics of participants who accepted the reception of messages

Characteristics	2-SMS (*n* = 32)	7-SMS (*n* = 28)	Total SMS (*n* = 60)	2-VMS (*n* = 49)	7-VMS (*n* = 51)	Total VMS (*n* = 100)	*p* value
Age							
Mean	48.1	48.1	48.1	47.7	48.1	47.9	0.90
Gender (%)							
Male	14 (43.8%)	12 (42.9%)	26 (43.3%)	23 (46.9%)	25 (49.0%)	48 (48.0%)	0.67
Female	18 (56.3%)	16 (57.1%)	34 (56.7%)	26 (53.1%)	26 (51.0%)	52 (52.0%)	
Health insurance plan (%)							
Subsidized	7 (21.9%)	6(21.4%)	13 (21.7%)	13 (26.5%)	14 (27.5%)	27 (27.0%)	0.51
Payroll	25 (78.1%)	22 (78.2%)	47 (78.3%)	36 (73.5%)	37(72.5%)	73 (73.0%)	
Education							
Elementary or lower	26 (81.3%)	19 (67.8%)	45 (75.0%)	37 (75.5%)	36 (70.6%)	73 (73.0%)	0.004
Secondary	-	2 (7.1%)	2 (3.3%)	8 (16.3%)	7 (13.7%)	15 (15.0%)	
Technician	5 (15.6%)	5 (17.9%)	10 (16.7%)	2 (4.1%)	6 (11.8%)	8 (8.0%)	
University	1 (3.1%)	2 (7.1%)	3 (5.0%)	2 (4.1%)	2 (3.9%)	4 (4.0%)	
Geographic region							
Andean	16 (50.0%)	16 (57.1%)	32 (53.3%)	26 (53.1%)	25 (49.0%)	51 (51.0%)	0.96
Caribbean	6 (18.8%)	4 (14.3%)	10 (16.7%)	8 (16.3%)	7 (13.7%)	15 (15.0%)	
Pacific	7 (21.9%)	4 (14.3%)	11 (18.3%)	10 (20.4%)	11 (21.6%)	21 (21.0%)	
Orinoquia and Amazonia	3 (9.4%)	4 (14.3%)	7 (11.7%)	5 (10.2%)	8 (15.7%)	13 (13.0%)	

*SMS* short message service (text), *VMS* voice message service; *2-SMS* two SMS weekly, *7-SMS* seven SMS weekly, *2-VMS* two VMS weekly, *7-VMS* seven VMS weekly

### Post-intervention Survey

One participant was lost to follow-up in the SMS arm and no losses to follow-up were observed in the VMS arm. All participants in the VMS arm declared having received at least one message during the last week of the intervention while in the 2-SMS and 7-SMS arms, only 56.2% and 89.3% declared having received at least one message, respectively (Table 3).

**Table 3:** Post-intervention participants and reception of messages

Study arm	Baseline participants	Lost to follow-up	Received at least one message*	Received expected number of messages*	Read/listened expected messages*	Mean amount of weekly messages received*
2-SMS	32	0	18 (56.2%)	10 (31.3%)	7 (21.9%)	0.9
7-SMS	28	1	25 (89.3%)	16 (59.3%)	10 (37.0%)	5.8
2-VMS	49	0	49 (100.0%)	48 (98.0%)	48 (98.0%)	3.1
7-VMS	51	0	51 (100.0%)	4 (7.8%)	4 (7.8%)	4.5
Total	160	1	144 (90.0%)	78 (48.7%)	69 (43.1%)	

*As declared by the participants. Expected messages as the expected number or higher

*SMS* short message service (text), *VMS* voice message service; *2-SMS* two SMS weekly, *7-SMS* seven SMS weekly, *2-VMS* two VMS weekly, *7-VMS* seven VMS weekly

With exception of the 2-VMS arm, all groups declared a mean number of weekly messages received lower than expected, and correspondingly, the percentage of participants declaring having read or listened the expected number of messages was below 50% (Table 3). Most participants found the messages useful, understandable, timely, and appropriate in their weekly frequency (Table 4). No significant differences were observed between arms; however, participants in the 2-VMS arms reported lower acceptability for the frequency of messages per week 36 out of 49 (73% and over) than participants in the remaining arms (83% and over). Despite the high acceptability for all criteria analyzed, 40.7% of participants in the SMS arm and 99.0% in the VMS arm declared in the post-intervention survey not being willing to be part of a future study with similar characteristics.

**Table 4:** Acceptability of phone messages for cancer prevention

Criteria	Response	2-SMS	7-SMS	Total SMS	2-VMS	7-VMS	Total VMS
Usefulness	Yes	17	24	41 (95.3%)	42	42	84 (84.0%)
	No	1	1	2 (4.7%)	7	9	16 (16.0%)
Understandability	Yes	18	25	43 (100.0%)	40	48	88 (88.0%)
	No	0	0	-	9	3	12 (12.0%)
Frequency	Yes	15	23	38 (88.4%)	36	44	80 (80.0%)
	No	3	2	5 (11.6%)	13	7	20 (20.0%)
Timing	Yes	15	25	40 (93.0%)	44	45	89 (89.0%)
	No	3	0	3 (7.0%)	5	6	11 (11.0%)
Acceptability of a future program	Yes	14	18	32 (54.2%)	0	0	0
	No	16	8	24 (40.7%)	48	51	99 (99.0%)
	Don’t know	2	1	3 (5.1%)	1	0	1 (1.0%)

*SMS* short message service (text), *VMS* voice message service; *2-SMS* two SMS weekly, *7-SMS* seven SMS weekly, *2-VMS* two VMS weekly, *7-VMS* seven VMS weekly

Only 2 out of 143 participants accessed the web portal as suggested in the phone messages. The most frequent reasons for not accessing the web portal were lack of time and lack of internet access (Table 5).

**Table 5:** Use of the web portal for cancer prevention

	Study arm				
Response	2-SMS	7-SMS	2-VMS	7-VMS	Total
A. Use of the web portal					
Yes	1	1	0	0	2 (1.4%)
No	17	24	49	51	141 (98.6%)
Total	18	25	49	51	143
B. Reasons for not using the web portal					
Forgot	1	2	7	5	15 (10.6%)
No internet access	6	13	11	13	43 (30.5%)
No time	8	7	16	16	47 (33.3%)
No interest	2	2	2	6	12 (8.5%)
Other reason	0	0	13	13	26 (18.4%)
Total	17	24	49	51	141

*SMS* short message service (text), *VMS* voice message service, *2-SMS* two SMS weekly, *7-SMS* seven SMS weekly, *2-VMS* two VMS weekly, *7-VMS* seven VMS weekly

## Discussion

Our study found low population response and participation in a pilot cancer prevention program based on mobile phone messages, with higher response rates for VMS worked by a dedicated company than SMS sent through the existing platform, but no differences in participation among responders.

Despite the promising results of randomized trials, the final impact of m-health would depend upon not only on efficacy of the intervention but also on their coverage and acceptability as determinants of active participation at the population level. However, most studies and reviews on m-health focus on efficacy in selected populations [17–19] and, in general, only a few population-based intervention studies report response and participation rates.

A study from India using text messages for diabetes control revealed a response rate lower than 1% after sending messages to over 100 million people [14]. These data substantially differ from acceptability surveys in Indian populations, where up to 86% declared their willingness to receive messages and 71.3% declared their willingness to use help lines [20]. Similarly, we found high acceptability on the use of phone messages for cancer prevention for all categories investigated among responders (Table 4), but low willingness to participate in similar studies in the future.

Moreover, the frequency of 2-VMS per week was the most consistent as per protocol with the declared number of messages received and listened by participants. In contrast, despite the high acceptability, the frequency of seven messages per week resulted in high recall of reception but low willingness to read/listen the messages, and the frequency of 2 SMS per week reported the lowest recall and willingness to read the messages (Table 3).

Differences between declared acceptability and practice highlight the need for more data derived from intervention studies (ideally population-based) but also suggest two major challenges to achieve impact of m-health at the population level: the contact as indicated by response rates and the willingness to participate. We found a 30% difference in response rates when using a dedicated company to reach the target population (VMS) compared with the regular platforms (SMS); however, once contacted, the willingness to participate revealed no significant differences between the two strategies, reducing the gap in participation rates to 9.5% (15.3% and 24.8%, respectively). Thus, a dedicated channel may improve participation; however, its use for massive communication is debatable and the magnitude of the effect deserves careful analysis contrasting the cost with the expected effects.

Acceptability of mobile phone messages has been widely investigated for interventions on different health conditions, as indicated, mostly through surveys and qualitative studies. Participation in m-health programs among populations with particular conditions could be higher including HIV, cancer, and other chronic conditions; current smokers; pregnant women; and overweight. Although any of these groups might be subject of preventive interventions, using m-health for behavioral change in the general population may face particular challenges. The lack of specificity for a given health condition could induce the perception of information overload [21], and some channels might be perceived as personal invasion [22]; these could be the reasons for the low willingness to be part of future programs in our study, particularly in the VMS arm. Previous reports have described other factors negatively associated with acceptability of SMS messages for health interventions in LMIC including lack of time (work conflict) [10] and psychosocial stressors such as caregiving roles and living in fear due to interpersonal violence and neighborhood insecurity [23].

Mobile phone messaging has been evaluated as a mean to help behavioral change through information on how to perform the desire behavior, information on barrier identification and problem solving, and information on how to set achievable goals [24]. Despite the theoretical background, interventions on health promotion such as increased physical activity or weight control have shown little to null effect [22, 24]; on the contrary, interventions for disease prevention such as immunization, screening, and sun protection have shown positive effects [24, 25], as well as interventions on populations with particular conditions as previously indicated.

Literacy (general and technological) has been described as one of the most frequent barriers for implementation of m-health in low- and middle-income countries [10]. Accordingly, we observed higher education (Technician/University) among affiliates who accepted participation in the study than affiliates in the general sample of the study (15.6% and 9.0%, respectively, *p* = 0.01), and higher education among those who accepted participation in the SMS arm than in the VMS arm (21.7% and 12.0%, respectively, *p* = 0.004). In addition, lack of internet access was the second most frequent reason for not visiting the web portal to search additional information (Table 5), suggesting lack of access to smartphones and other mobile technologies as a limitation for online apps and programs.

Population-based surveys on m-health acceptability from Asia and Africa have also found younger age, female gender, employment, and higher socioeconomic condition positively associated with willingness to receive mobile phone health messages [13, 26]. We found no significant differences in age, gender, and health insurance plan neither between the general sample and participants nor between the SMS and VMS arms; however, no-responders were more frequently men. Participants were also less frequently men but less frequently affiliated to the subsidized health insurance as compared with the population in the general sample of the study (Tables 1 and 2).

To our knowledge, this is the first study conducted in the Latin American region to assess acceptability of mobile phone messages for prevention of a chronic condition. Some experiences have been implemented aimed at improving quality of care in diabetes, hypertension, and pregnancy [27–29]. Specific studies on preventive interventions would help identify barriers and determinants of successful m-health programs in the region including differences in cultural backgrounds (rural, urban, indigenous) and health system characteristics. We found no differences between regions of the country but no investigation was done on other factors related with population backgrounds. In addition, the high insurance coverage of the Colombian health system may determine lower awareness and willingness to adopt preventive practices as compared with fragmented health systems existing in several Latin American countries where concerns about cancer prevention might be higher.

The study results suggest a limited population impact of m-health for cancer prevention messages given low participation rates, little willingness to read/listened messages for frequencies other than 2-VMS per week, and the gap between the declared acceptability and practice. However, participation between 15 and 25% also suggests a potential role in public health practice if mobile phone messages for cancer prevention are part of comprehensive multicomponent and multilevel interventions to achieve behavioral change, and by no means conceiving m-health as the sole intervention or a substitute of education by regular preventive services.

### Study Limitations

Although our sample was randomly selected from a population of affiliates to the Colombian health system, the inclusion of only one health insurance company limits the generalization of the results to the whole Colombian population. Nevertheless, the health insurance company included in the study is one of the biggest in the country with representation of all regions and the two existing health insurance plans in the public system.

The response to the recruitment call was part of the outcomes to be measured given its final impact on participation rates; hence, no replacement or adjustment was planned for non-responders. With the exception of gender, we found no differences between responders and non-responders to the recruitment call. Since non-responders were more frequently men, the results should be interpreted with caution regarding this variable.

Despite the limitations, we consider our study provides relevant information on the potential of m-health for cancer prevention at the population level in a region with scarce information on the subject, and accordingly, it gives a relevant input for a proper design of communication strategies aimed at reducing the burden of disease.

## Data Availability

All data and materials comply with field standards.

## Appendix. Messages sent via SMS and VMS

### English translation (back)

Messages were sent in Spanish (corresponding version below), based on a free translation and adaptation from the original messages of the European Code Against Cancer (4th edition) in its English version. SMS and VMS were identical. “Nueva EPS” corresponds to the health insurance company.

- Cancer prevention. Do not smoke. Do not use any form of tobacco. Nueva EPS. More information at http://prevencion.iarc.fr
- Cancer prevention. Make your home smoke free. Nueva EPS. More information at http://prevencion.iarc.fr
- Cancer prevention. Maintain a healthy weight. Nueva EPS. More information at http://prevencion.iarc.fr
- Cancer prevention. Exercise every day. Limit the time you spend sitting. Nueva EPS. More information at http://prevencion.iarc.fr
- Cancer prevention. Eat plenty of whole grains, pulses, fruits and vegetables. Nueva EPS. More information at http://prevencion.iarc.fr
- Cancer prevention. Limit foods high in sugar or fat and avoid sugary drinks. Nueva EPS. More information at http://prevencion.iarc.fr
- Cancer prevention. Avoid processed meat; limit red meat and foods high in salt. Nueva EPS. More information at http://prevencion.iarc.fr
- Cancer prevention. Limit alcohol intake. Not drinking alcohol is better for cancer prevention. Nueva EPS. More information at http://prevencion.iarc.fr

Recommendation for reading the URL of the web portal in voice messages:

More information at aitch, tee, tee, pee, colon, slash, slash, prevention, period, i, ay, ar, cee, period, eff, ar.

### Spanish version

- Prevención de cáncer. No fume. No consuma ningún tipo de tabaco. Nueva EPS. Mas información en http://prevencion.iarc.fr
- Prevención de cáncer. Haga de su casa un hogar sin humo. Nueva EPS. Mas información en http://prevencion.iarc.fr
- Prevención de cáncer. Mantenga un peso saludable. Nueva EPS. Mas información en http://prevencion.iarc.fr
- Prevención de cáncer. Haga ejercicio a diario. Limite el tiempo que pasa sentado. Nueva EPS. Mas información en http://prevencion.iarc.fr
- Prevención de cáncer. Consuma gran cantidad de cereales integrales, legumbres, frutas y verduras. Nueva EPS. Mas información en http://prevencion.iarc.fr
- Prevención de cáncer. Limite los alimentos ricos en azúcar o grasa y evite las bebidas azucaradas. Nueva EPS. Mas información en http://prevencion.iarc.fr
- Prevención de cáncer. Evite la carne procesada; limite el consumo de carne roja y de alimentos con mucha sal. Nueva EPS. Mas información en http://prevencion.iarc.fr
- Prevención de cáncer. Limite el consumo de alcohol, lo mejor para prevenir el cáncer es evitarlo del todo. Nueva EPS. Mas información en http://prevencion.iarc.fr

Sugerencia de lectura del URL del portal de internet en los mensajes de voz:

Más información en hache, te, te, pe, dos puntos, barra inclinada dos veces, prevención, punto, i, a, ere, ce, punto, efe, ere
